# What proportion of couples with a history of recurrent pregnancy loss and with a balanced rearrangement in one parent can potentially be identified through cell-free DNA genotyping?

**DOI:** 10.1186/s13039-023-00657-x

**Published:** 2023-09-29

**Authors:** Laura J. C. M. van Zutven, Jona Mijalkovic, Monique van Veghel-Plandsoen, Margaret Goense, Marike Polak, Maarten F. C. M. Knapen, Sabina de Weerd, Marieke Joosten, Karin E. M. Diderich, Lies H. Hoefsloot, Diane Van Opstal, Malgorzata I. Srebniak

**Affiliations:** 1 Department of Clinical Genetics, Erasmus Medical Centre, Dr Molewaterplein 40, 3015 GD Rotterdam, The Netherlands; 2 Department of Psychology, Education & Child Studies (DPECS), Erasmus University, Rotterdam, The Netherlands; 3 Department of Obstetrics and Prenatal Medicine, Erasmus Medical Centre, Wytemaweg 80, Na-1517, 3015 GE Rotterdam, The Netherlands; 4 Department of Obstetrics and Gynaecology, Albert Schweitzer Hospital, Albert Schweitzerplaats 25, 3318 AT Dordrecht, The Netherlands

**Keywords:** Recurrent pregnancy loss, NIPT, Karyotyping, Balanced translocation, cfDNA

## Abstract

**Background:**

Balanced chromosome aberrations are reported in about 1:30 couples with recurrent pregnancy loss (RPL). Karyotyping of both parents is necessary to identify these aberrations. Genome-wide non-invasive prenatal testing (NIPT) in case of recurrent pregnancy loss could be a more efficient way to identify couples at increased risk for carrying a balanced chromosome rearrangement. The aim of this study was to evaluate whether the potential fetal imbalances caused by parental balanced aberrations detected in our center are large enough to be detectable by genome-wide non-invasive prenatal testing (NIPT).

**Material and methods:**

From January 1970 until May 2020 our laboratory received 30,863 unique requests for karyotyping due to RPL. We have identified 16,045 couples and evaluated all abnormal cytogenetic results to assess the minimal size of the involved chromosomal segments in potential unbalanced products of the rearrangements.

**Results:**

In the presented cohort we detected 277 aberrant balanced translocations/inversions in females and 185 in males amongst 16,045 couples with RPL, which can be translated to a risk of 1:35 (2.9%, 95% CI 2.6–3.2%). Our study showed that the vast majority (98.7%, 95% CI 97.1–99.5%) of these balanced aberrations will potentially cause a fetal imbalance > 10 Mb, which is detectable with genome-wide NIPT if it was performed during one of the miscarriages.

**Conclusions:**

Our study suggests that genome-wide NIPT is able to reveal most unbalanced products of balanced chromosomal rearrangements carried by couples with RPL and therefore can potentially identify balanced chromosomal aberration carriers. Moreover, our data suggest that these couples can be offered NIPT in case they decline invasive testing in future pregnancies.

## Introduction

Recurrent pregnancy loss (RPL), defined as two pregnancy losses prior to 20 weeks, occurs in 1–3% of all couples [1]. The well-known factor causing RPL is the presence of fetal unbalanced chromosomal aberrations, but even after comprehensive investigations according to current standards, the etiology of RPL is identified in fewer than 50% of couples [2]. Being a carrier of a balanced structural chromosomal aberration not only leads to a risk for RPL, but depending on the breakpoints, there is also an increased risk for unbalanced offspring with congenital anomalies and/or intellectual disability. This risk is highly individual and depends on the unique translocation breakpoints. Balanced (reciprocal) translocations are reported in about 1:500 unselected individuals, but are detected in about 1:30 couples with a reproductive history of miscarriages [3]. Until recently, in the Netherlands time-consuming karyotyping in parental blood or investigations of miscarriage tissue were the only techniques to detect chromosomal aberrations in such couples. Many authors have previously shown that the microarray technique when applied on the product of conception successfully overcomes problems with cell culture and detects maternal contamination [4]. However, although microarray testing is the method of choice, products of conceptions (POC) aren’t always available for testing or aren’t of the desired quality [5]. Because of this, we search for a testing method based on cell-free DNA (cfDNA) in maternal plasma, as maternal blood could be sampled at the moment a miscarriage is confirmed by early ultrasound. Nowadays, genome-wide non-invasive prenatal testing (NIPT) using maternal plasma cfDNA offers an alternative to diagnose an unbalanced product of a parental translocation for couples who decline to undergo invasive testing [6–8]. Therefore, we wondered whether it would be feasible to offer genotyping of cfDNA during the miscarriage to identify the derivative chromosomes and therefore potential parental carriers of a balanced structural rearrangement, who can then be karyotyped, instead of karyotyping all couples after RPL. To achieve this, it is necessary that potential imbalances are large enough (> 10–15 Mb) to be detectable with genome-wide NIPT. Thus, we have evaluated the cytogenetic results in a large cohort of parents karyotyped due to RPL in our laboratory. We determined the length of the chromosomal segments involved in the structural rearrangement and we investigated how many of the unbalanced parental translocations and inversions could be detected through cfDNA genotyping in case of a miscarriage.

## Material and methods

**Fig. 1:**
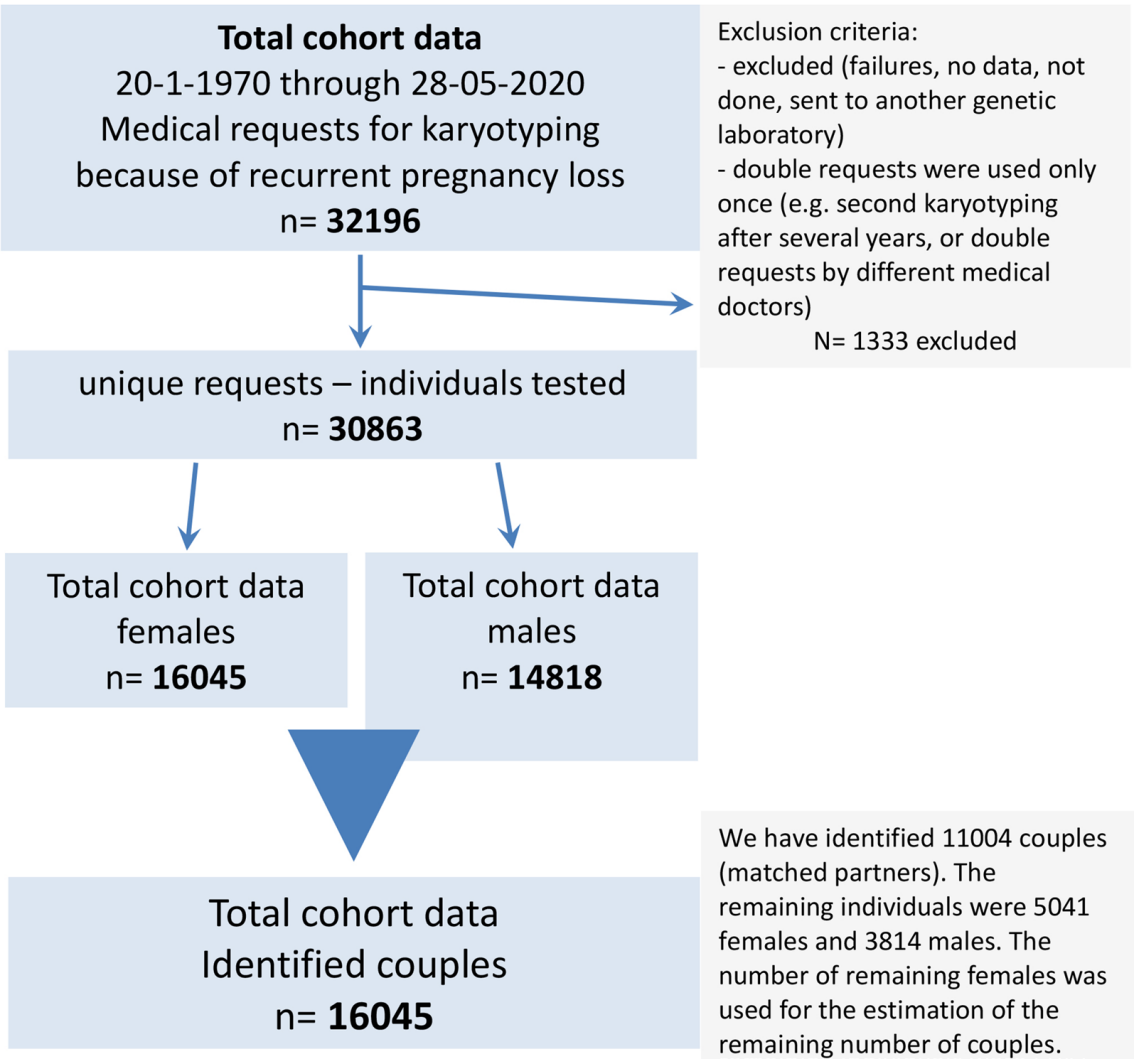
Cohort selection

From January 1970 until May 2020 our laboratory received 32,196 requests for karyotyping because of RPL. Out of these data we have extracted 30,863 unique requests (Fig. 1). To be able to calculate the prevalence of balanced translocations and inversions per couple, we have identified 11 004 couples (matched partners). The remaining individuals were 5041 females and 3814 males. The number of females was used for the estimation of the number of the remaining couples, so we do not have data on males in at least 1227 (7.6%) couples. Therefore we concluded that we have investigated a maximum number of 16,045 couples based on the number of females in the cohort. The data represent a heterogeneous cohort with RPL, where number of miscarriages is not stated on the request form. It was therefore not analyzed for other factors contributing to the risks of RPL.

To determine how many fetuses with an unbalanced familial chromosomal aberration in the present cohort would be detected by NIPT, we have reviewed all cases of balanced translocations and inversions to theoretically assess the size of potential imbalances in the fetus. When the potential imbalance is large enough, the fetal fraction is sufficient and pregnancy loss is recognized before the tissues are discharged from the uterus, such a patient could be offered NIPT. The resolution of NIPT in the Netherlands is ~ 10–15 Mb based on the use of WISECONDOR as described before [9]. Additionally, since 2021 the VeriSeq NIPT Assay Software is used for NIPT analysis and has a resolution of 7 Mb [8, 9]

Because the translocation/inversion breakpoints were determined with karyotyping, the actual breakpoints at the molecular level are not known. To estimate the size, we considered the smallest possible segmental imbalances by using the chromosomal band boundary closest to the telomere. The segment size estimation utilized the chromosome band boundaries in GRCh37/hg19. For instance, when a translocation involved band 16q22, its chromosomal location is chr16:66,700,001–74100000. The minimal segment size is estimated in the following way: the 16q-telomere position at chr16: 90,354,753 minus 74,100,000 which is the band boundary closer to the telomere, are giving the minimal size of 16 Mb. Chromosomes with a breakpoint in a terminal chromosome band, were assessed to produce a minimal unbalanced region of nearly 0 Mb and were classified as not detectable.

The derivative chromosome was considered potentially detectable if the size of at least one of both chromosomal segments (deleted or duplicated) was above the currently used NIPT resolution. We assigned the possible imbalances to the following categories:

- NIPT recommended if the largest segment ≥ 15 Mb
- NIPT challenging if the largest segment is 10–15 Mb
- NIPT very challenging if the largest segment is 7–10 Mb
- NIPT not possible if both unbalanced regions have a minimal size < 7 Mb

All theoretical unbalanced segregation patterns were considered (adjacent 1 and 2, and 3:1), irrespective of the sizes of the imbalances, although according to the literature alternate and 2:2 adjacent-1 are the most commonly seen (93.2%) [8] and some of the other segregation patterns may not be empirically encountered, because they involve too large chromosomal segments that are non-viable. If one of the potential imbalances was larger than the threshold, such a translocation was considered detectable. Unbalanced translocations with breakpoints less than 7 Mb from the telomere were considered to be non-detectable although theoretically a 3:1 mal-segregation will lead to products with imbalances larger than 15 Mb. This is because of the rarity of 3:1 mal-segregation in such translocation type [10] and if 3:1 occurs, it will probably never reach the gestational age at which NIPT can be performed.

The recurrent t(11;22)(q23;11.2) causing Emanuel syndrome through 3:1 segregation resulting with an extra derivative chromosome+der(22)t(11;22) (OMIM 609029), is detectable through NIPT, as shown previously, because the 11q imbalance is ca. 18 Mb [8]. However, if the potential imbalance on chromosome 11 would be smaller than 7 Mb in such a case, the presence of an extra small derivative chromosome with both an undetectable imbalance of the acrocentric chromosome and an undetectable imbalance of the partner chromosome would be missed.

Pericentric inversions can form a recombination chromosome with a deleted and a duplicated segment. Therefore if the minimal size of one of these segments is large enough such a recombinant chromosome is potentially detectable by NIPT if there is sufficient fetal fraction and the pregnancy loss did not occur before NIPT could be done. The common pericentric inversions inv(2)(p11.2q13) and inv(9)(p12q13) are recognized as normal variants (ISCN 2020) and therefore were not reported in karyotyping results and thus excluded from this study.

Paracentric inversions are typically not an indication for invasive prenatal testing, as the recombinants are dicentric and acentric chromosomes and the unbalanced products of conception are empirically almost never encountered in miscarriage tissues [9]. Therefore, we assumed that they would theoretically be larger than 15 Mb and thus theoretically detectable. However, unless NIPT can be performed very early in pregnancy, we do not expect to find these kind of imbalances.

### Statistical analysis

The percentage of cases is reported with Agresti–Coull 95% confidence intervals, which have a good coverage probability for larger samples (*n* > 40) [11]. Categorical data were compared using the chi-square test or Fisher exact test, depending on the outcome frequency. Computations were performed using the Epitools epidemiological calculator (Sergeant, ESG, 2018. Epitools Epidemiological Calculators. Ausvet. Available at: http://epitools.ausvet.com.au).

## Results

**Fig. 2:**
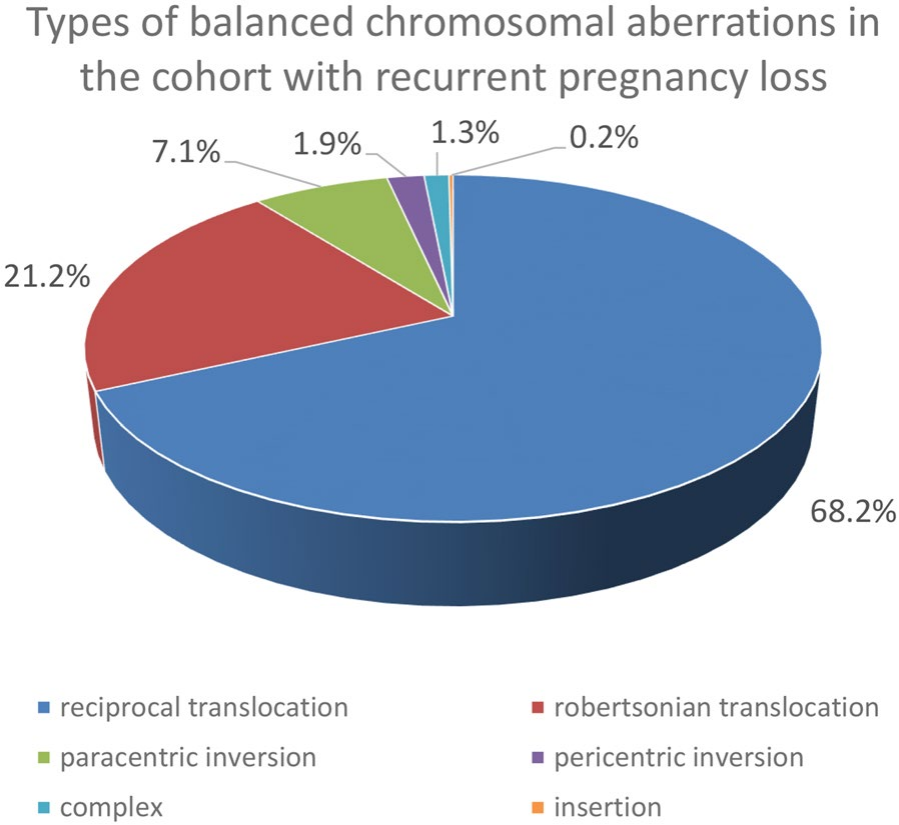
Types of balanced chromosomal aberration in the individuals karyotyped due to recurrent pregnancy loss

In the cohort of 16,045 couples we have detected 277 balanced chromosome aberrations in females (277/16045 or 1.73%) and 185 aberrations in males (185/14818 or 1.25%). Therefore we concluded that there were 462 balanced translocations/inversions found in 16,045 couples with recurrent miscarriages, which can be translated to the risk of 1:35 (2.9%, 95% CI 2.6%-3.2%) per couple (Table 1). Figure 2 shows the types of balanced chromosomal aberrations found in our cohort. Females more often carried balanced chromosomal aberrations than males (*p* < 0.001). Although males (132/185 or 71.4%) more often carried unique reciprocal translocations than females (183/277 or 66.1%), this difference was not statistically significant.

**Table 1:** Rearrangement types and risks in the cohort with recurrent pregnancy loss (RPL) in males, females and the whole karyotyped cohort

Rearrangement types and risks in the cohort with RPL	Number of aberrations in males	Percentage (%) aberrations in males	Number of aberrations in females	Percentage (%) aberrations in females	*p*-Value*	Total number of aberrations	Percentage
Reciprocal translocation	132	71.4	183	66.1	0.232	315	68.2
Robertsonian translocation	31	16.8	67	24.2	0.056	98	21.2
Paracentric inversion	16	8.6	17	6.1	0.304	33	7.1
Pericentric inversion	3	1.6	6	2.2	0.747	9	1.9
Complex	2	1.1	4	1.4	1.00	6	1.3
Insertion	1	0.5	0	0	0.400	1	0.2
Total	185	**1.25%** (185/14818 individuals)	277	**1.73%** (277/16045 individuals)	< 0.001	462	**2.88%** (462/16045 couples)
Risk for balanced rearrangement causing RPL		**1:80** in males		**1:58** in females			**1:35** in couples

Bold value signifies risk figures

*p-Value for the difference between percentages aberrations in males versus females, where chi-square test for proportions was used or Fisher’s exact test (2-sided) for expected counts < 5

As most of the aberrations had unique breakpoints (all except Robertsonian translocations) the minimal size of unbalanced regions was calculated individually for all carriers. In the presented cohort there were no cases of reciprocal translocations that could produce undetectable imbalance resulting from 3.1 segregation type. This kind of imbalance would rather result in a viable pregnancy with an affected fetus than in recurrent miscarriage [9].

**Fig. 3:**
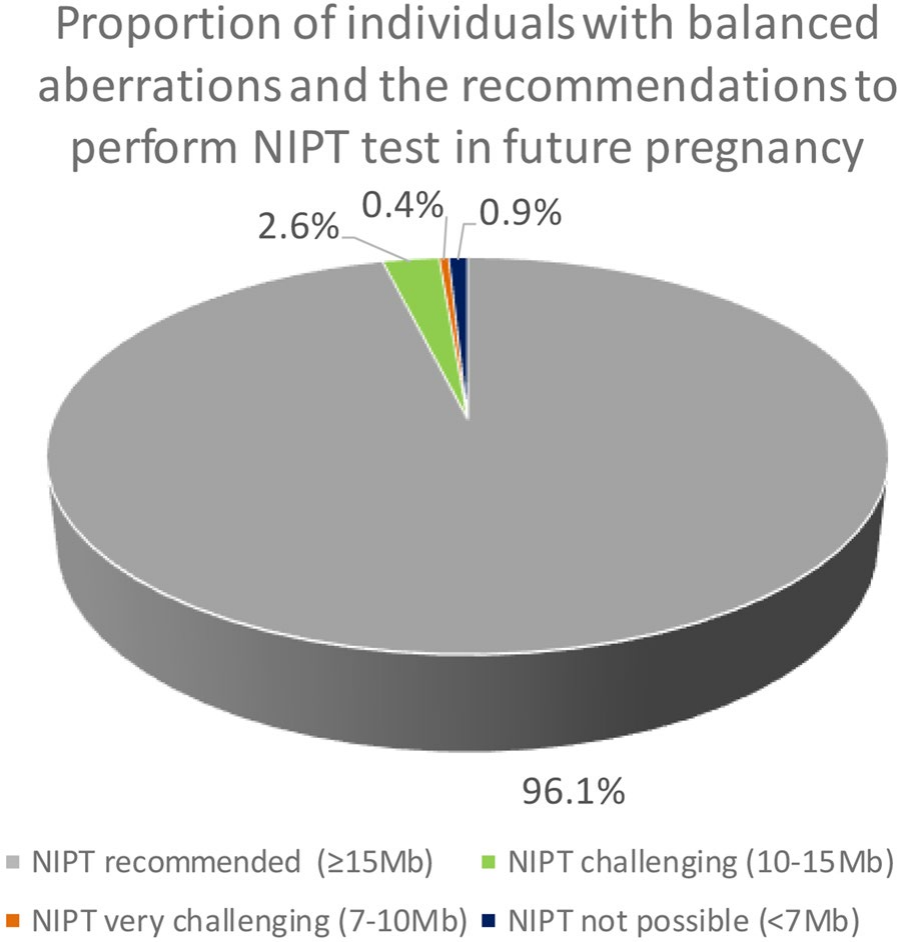
Proportion of individuals with balanced aberrations and the recommendations on NIPT in future pregnancies. The recommendation is based on the assessment of the minimal size of the potential imbalance. The minimal size of the largest segment was used to categorize the patients

Based on the minimal size of the largest of both segmental imbalances, we estimated whether such a carrier could be identified through NIPT in a pregnancy of an unbalanced conception (either ongoing or at the time of miscarriage) and whether the couple/individual could be offered NIPT in future pregnancies. The results are illustrated in Fig. 3. In 444/462 or 96.1% (95% CI 93.9–97.6%) of the cases, the parental balanced aberration potentially leads to at least one chromosomal imbalance larger than 15 Mb. These were assessed as detectable by NIPT and in such cases, NIPT could be recommended. In 2.6% of patients, the largest of both potential imbalances would be between 10 and 15 Mb and in 0.4% the minimal size was between 7 and 10 Mb. These were both assessed as detectable by NIPT (this is the official resolution of the VeriSeq NIPT solution v2), but either challenging or very challenging with prior information on the parental karyotype potentially being crucial for correct interpretation of NIPT in these cases. In 0.9% of the carriers, both segmental imbalances were smaller than 7 Mb and therefore classified as undetectable with NIPT. Therefore, the vast majority (456/462 or 98.7%, 95% CI 97.1–99.5%) of these balanced aberrations could potentially cause a fetal imbalance > 10 Mb, which is detectable through genome-wide NIPT test if it was performed during one of the miscarriages.

## Discussion

Balanced (reciprocal) translocations are reported in a significant proportion of couples with RPL. Although it is thought that Robertsonian translocations are common, we showed that reciprocal translations with unique breakpoints (68.2% of all aberrations) are more often detected. Therefore a genome-wide analysis needs to be performed to detect those unique structural variants. Although new molecular methods are emerging, at this moment the only widely implemented technique to detect balanced translocations and inversions in clinical settings is laborious karyotyping. To ensure the efficiency of testing, clinicians attempted to select couples for karyotyping. However, a recent review found insufficient evidence for a difference in the frequency of abnormal karyotyping results between women with two and three or more pregnancy losses [12]. As karyotyping is a laborious method, we searched for an alternative high throughput method to identify potential carriers after recurrent miscarriages have been diagnosed, ideally a method that could also be used for prenatal screening in future pregnancies.

Our study shows that the vast majority of couples carrying a structural chromosome aberration could potentially be identified if cfDNA genotyping (genome-wide NIPT) was employed at the time of pregnancy loss after 8 weeks of pregnancy: 98.7% of couples carried a balanced aberration potentially causing at least one imbalance larger than 10 Mb. However, although adjacent 1 and 2 segregation patterns are most common, in clinical settings, while assessing individual patients' elibility for the NIPT test one needs to take also 3:1 mal segregation into account. Especially if an acrocentric chromosome is involved, it may produce smaller imbalance than the typical adjacent segregation pattern.

The feasibility to detect unbalanced translocations through cfDNA (NIPT testing) has already been shown by several authors [7, 8, 13, 14]. However, a risk of missing an unbalanced aberration due to NIPT limitations such as too low fetal fraction (sometimes due to high BMI) and limited resolution should be included in the pre-test counseling if NIPT was offered for this purpose.

So far, there have been only a few studies that investigated cell-free DNA testing in nonviable gestations. Several studies showed that cell-free fetal DNA persists in the maternal plasma when the gestation remains in situ, possibly as a result of continuous placental apoptosis after fetal demise [15–17]. Therefore, blood sampling for cfDNA genotyping at that point is a good alternative for identifying the chromosomal aberrations causing pregnancy loss.

### Additional benefits of NIPT testing in miscarriage

Another argument for performing NIPT in case of a miscarriage is the fact that it would not only lead to the identification of potential balanced aberration carriers, but it will also identify other chromosomal causes of early miscarriage. Chromosomal abnormalities are found in ca. 50% of fetal deaths at less than 20 weeks of gestation and 6–15% at more than 20 weeks of gestation [18–21]. The American Society of Reproductive Medicine recommends cytogenetic evaluation of the products of conception in women with two or three spontaneous miscarriages [22]. Still, so far there is no such recommendation in the Netherlands. Detecting the cause of fetal loss may not only prevent other unnecessary testing in case of RPL [14], but it will probably also positively contribute to the psychological coping process after RPL [23]. It has been shown that an abnormal embryonic karyotype is the most frequent cause of recurrent miscarriage and the true unsolved cases can be limited to about 25% of the couples with RPL [24]. Peng et al. recently showed that in comparison to current practices of cytogenetic testing of products of conception (POCs), cfDNA testing allowed not only for a lower cost per patient but for better sample accessibility as well: blood sampling for cfDNA test immediately after diagnosing a non-viable pregnancy is possible. In addition, using cfDNA can potentially reduce the number of patients undergoing unnecessary workups resulting in overall cost savings [14]. The study of Peng and colleagues showed that the inclusion of cfDNA testing is a cost-effective approach to guide RPL workup and not only to identify balanced translocation carriers.

### NIPT in future pregnancies

Finally, an additional advantage is that a cfDNA test (NIPT) can be employed in future pregnancies as an alternative to invasive testing, when a couple was shown to carry a balanced chromosome aberration that NIPT can detect if the fetus is unbalanced. We have previously shown that unbalanced translocations are detectable in maternal plasma cfDNA, and assessed that imbalances larger than 10 Mb should be detectable if the fetal fraction is sufficiently high [8].

Genome-wide NIPT testing in future pregnancies could be recommended to couples with RPL to investigate the presence of unbalanced translocation/inversion, even if testing of a previous pregnancy loss failed or cfDNA test was unavailable or failed due to too low fetal fraction. Additionally, it should be noted that the NIPT test is a high throughput technique where patient samples can be run in multiplex reactions, whereas parental karyotyping is still a rather laborious process, resulting in more expensive hands-on time per patient than in case of NIPT analysis. We anticipate that testing the pregnancy loss with NIPT instead of directly karyotyping both partners in cases of RPL might be more (cost)efficient [14]. It will allow selecting only those parents at risk for being a carrier instead of karyotyping all parents with RPL. Moreover, it will help to diagnose the etiology of pregnancy loss in more cases [25]. However, it should be noted that high throughput SNP microarray testing in POC is the method of choice if the miscarriage tissue is available and of desired quality.

### Limitations of the NIPT approach

The conclusions of this study are based on the theoretical assessment of the size of the chromosomal segments, irrespective of viability of the different unbalanced patterns Many segregation products will have very large imbalances that would be lethal and not reach the gestational age when NIPT can be performed. The cfDNA test depends on the moment of the blood collection and the fetal cfDNA fraction present. It should be drawn as soon as fetal death is diagnosed when the pregnancy is still in situ. Otherwise, some patients may be too late for blood collection. Some patients who experience a miscarriage before 8 weeks of gestation may not benefit from the NIPT approach as before 7 weeks of gestation the fetal fraction of cfDNA is more likely to be insufficient [16]. Although most spontaneous miscarriages are detected between 8 and 13 weeks of gestation, couples with recurrent miscarriages are very aware of becoming pregnant and early monitoring can lead to earlier detection of the miscarriage [16]; therefore, blood can be drawn at the moment of diagnosis before the products of conception are discharged [5, 26]. Colley and colleagues have shown that in 66% of samples before 7 weeks of gestation their cfDNA test provided successful diagnosis [16]. cfDNA testing cannot replace karyotyping or microarray in fetal tissue, however it has potential to detect the cause of miscarriage in couples with RPL. Potentially in about 6% of patients a retention of the trophoblastic tissue can be observed [27], however, to our knowledge there are no reports on usefulness of cfDNA in such cases. In this study, we analyzed only the size of the theoretical, potential imbalances and the possibility of its detection assuming the blood sample contains enough fetal cfDNA. Large prospective cohort studies are necessary to establish how many patients cannot benefit from the cfDNA test, either because the sampling is performed too late or the miscarriage occurred before 8 weeks of pregnancy and insufficient fetal fraction did not allow to achieve NIPT results.

## Conclusions

Our study suggests that genome-wide NIPT is able to detect unbalanced conception products in the vast majority of couples with RPL and balanced chromosomal aberrations (98.7%) and therefore can identify potential balanced chromosomal aberration carriers. Moreover our data suggest that these couples can be offered NIPT in case they decline invasive testing in future pregnancies.

## Data Availability

The datasets used and/or analyzed during the current study are available from the corresponding author on reasonable request.

## Abbreviations

cfDNA: Cell free DNA
RPL: Recurrent pregnancy loss
NIPT: Non-invasive prenatal testing
